# Influence of a Bacteriophage Cocktail as a Biocontrol Strategy on Sulfate-Reducing Bacteria in a Pilot-Scale System

**DOI:** 10.3390/bioengineering13101166

**Published:** 2026-10-07

**Authors:** Marcella Silva Vieira, Roberto Sousa Dias, Helena Santiago Lima, Maíra Paula de Sousa, Cynthia Canêdo da Silva, Sérgio Oliveira de Paula

**Affiliations:** 1 Department of Microbiology, Federal University of Viçosa, Viçosa 36570-900, Minas Gerais, Brazil; marcellav46@gmail.com (M.S.V.); hsantiagolima@gmail.com (H.S.L.); ccanedosilva@gmail.com (C.C.d.S.); 2 Department of General Biology, Federal University of Viçosa, Viçosa 36570-900, Minas Gerais, Brazil; rosousa318@gmail.com; 3 Leopoldo Américo Miguez de Mello Research and Development Center (CENPES), Petrobras, Rio de Janeiro 20230-010, Brazil; mpsousa@petrobras.com.br

**Keywords:** pilot tanks, bacteriophages, biocontrol, microbial diversity

## Abstract

Sulfate-reducing bacteria (SRB) play crucial roles in microbiologically induced corrosion (MIC), especially in anaerobic environments where the production of hydrogen sulfide (H_2_S) and biofilm formation accelerate metal degradation. Conventional treatments, such as THPS-based biocides, while effective, face limitations relating to the development of resistance and poor biofilm penetration. In this study, we evaluated the use of a non-specific bacteriophage cocktail as a biological control strategy against a mixed SRB culture (AP) biofilm and related corrosion, utilizing a pilot-scale system with AISI 1020 carbon steel coupons. Over 38 days, we monitored H_2_S production, optical density, and ATP concentrations, along with the surface characterization of steel coupons through scanning electron microscopy (SEM) and profilometry. Metagenomic sequencing revealed changes in microbial community composition, including reductions in sulfate consumers and shifts in dominant taxa. SEM and profilometric analyses showed disrupted and less aggressive biofilms on treated surfaces, often exhibiting filamentous structures. Diversity indices indicated the modulation of microbial richness and composition over time. These findings demonstrate the potential of bacteriophage cocktails to reshape complex microbial communities and reduce MIC, providing a sustainable alternative to chemical biocides in large-scale industrial settings.

## 1. Introduction

Microorganisms are intimately connected to changes that happen daily in the environment, even when unseen. They are ubiquitously present across the planet, occupying diverse ecological niches that range from soil, water, and air to the human body itself, where they form complex microbiomes [1,2]. Some examples are provided by sulfate-reducing prokaryotes, which play a role in the sulfur cycle, drain acid mines, and are responsible for microbiologically induced corrosion (MIC) [3,4,5]. This enormous group encompasses microorganisms from both domains, Archaea and Bacteria, with the latter being the most representative and diverse, comprising more than 220 species across 60 genera. Sulfate-reducing bacteria (SRB) belong to the Proteobacteria phylum, Deltaproteobacteria class, organized into three groups: thermophiles (genus *Thermodesulfobacterium*), spore-forming Gram-positives (genus *Desulfotomaculum*), and the largest group, mesophilic Gram-negatives (which include genera as *Desulfobacterium* and *Desulfovibrio*) [6,7].

Economic losses due to metal corrosion are notable in the oil and gas industry, varying around US$2.5 trillion, and SRB play an important role by consuming the sulfur and carbon sources available in an anaerobic environment [8]. During anaerobic respiration, dissimilative sulfate reduction is responsible for generating hydrogen sulfide (H_2_S), a corrosive and toxic gas that can act directly and indirectly on metal, such as carbon steel. Besides H_2_S, biofilm formation (a community formed by sessile cells adhered to a surface and immersed in an extracellular polymeric substance, EPS) contributes to accelerating the corrosive process through a positive interaction between metal and SRB, since that material provides sources for metabolism. MIC is currently perceptible as crevice corrosion, cracking, and dealloying, but is most found as pitting morphology [9,10].

Bacteriophages (“phages”) are viruses that specifically infect bacteria and have been highlighted as a promising alternative for microbial control [11,12]. They represent a crucial solution to the challenge of antibiotic resistance, with established applications in human and veterinary medicine, food industry, agriculture, biotechnology, and pharmacy [13]. Particularly, lytic phages that carry depolymerases and endolysin enzymes can degrade complex biofilm structures [14,15,16], and those lytic genes have been utilized in engineered bacterial platforms as novel delivery systems for targeted oncological therapies [17]. However, concerning the SRB group, their taxonomic heterogeneity and the technical challenges associated with their required anoxic environments make the use of non-specific phages an advantageous strategy. The ability of these viruses to disrupt the EPS matrix, potentially without requiring a productive infection of the target host, is one of the mechanisms for biofilm mitigation [18,19,20].

Conventional methods for mitigating MIC frequently involve the application of chemical biocides such as tetrakis(hydroxymethyl)phosphonium sulfate (THPS) [21,22]. Another method that has been tested includes biomineralization, cathodic polarization, and the addition of alloying elements [23]; however, bacterial sessile cells (biofilm formation) may require higher dosages of biocide, further developing plasticity to acquire resistance after several treatments [24]. THPS concentrations can range from 50–100 ppm to 500–1000 ppm to eliminate the cells, and the sulfate released from biocide dissociation can stimulate SRB growth [25,26]. Another concern is how THPS acts on the deepest cells in biofilms, where its diffusion into this structure is slower. While other eco-friendly strategies, such as biocide enhancers (peptides) or plant extracts to disrupt the biofilm matrix have been explored, they still present operational limitations, including susceptibility to rapid degradation and challenges regarding scaling up to large industrial volumes [27,28,29,30].

The formulation of phage cocktails (solutions containing two or more viruses) is an efficient method to degrade multi-species biofilms [31]. This approach is particularly relevant as multi-species communities are the most encountered form in the environment, including within the SRB community [32,33,34]. In addition, the use of cocktails expands the spectrum of desirable effects and overcomes the inherent limitations of using a single phage as the emergence of bacterial resistance [35,36]. As an eco-friendly method, the application of phages is a promising and continuously advancing strategy for industrial sectors, sustained by clear operational advantages. Phages offer low production costs and high accessibility, as isolation sources can be derived from matrices such as wastewater, and additionally, these viruses are under numerous studies under different conditions of temperature and salinity, enabling prolonged application and robust formulations [37,38]. Within complex industrial biofilms, the core distinction of this method lies in its potential to complement chemical strategies by providing long-term protection through the persistent enzymatic disruption of the extracellular polymeric matrix, even under dynamic large-scale conditions.

The aim of the present study was to investigate and describe the effects of a cocktail of nonspecific bacteriophages aiming for the control of biofilm and H_2_S, triggered by a mixed culture of SRB (herein called AP, from “produced water”). This work proposes an approach beyond conventional microplate assays, involving the experimental use of a pilot tank system with an 8 L capacity, which has carbon steel coupons AISI 1020 coupled to its structure for MIC assessment. The analysis of SEM, profilometry, corrosion rate, and metagenome sequencing contributed to understanding the behavior of the mixed culture AP and the cocktail.

## 2. Material and Methods

### 2.1. SRB Cultivation

The SRB mixed culture AP was an environmental consortium recovered from petroleum produced water. To ensure a functional population of SRB, the sample was cultivated in modified Postgate E medium (KH_2_PO_4_ 0.5 g/L, NH_4_Cl 1 g/L, Na_2_SO_4_ 1 g/L, CaCl_2_·6H_2_O 1 g/L, MgCl_2_·6H_2_O 1.83 g/L, yeast extract 1 g/L, ascorbic acid 0.1 g/L, sodium lactate 50% (*m*/*v*) 7.0 mL, resazurin 0025% (*m*/*v*) 4.0 mL, pH 7.6) prepared with natural seawater. The pre-inoculum was composed of 10% (total volume 50 mL) and stored in a bacteriological stove at 30 °C for seven days. After that, an inoculum of 400 mL was produced in a Schott flask containing 40 mL of SRB, which was stored in an anaerobic chamber (Whitley A95^®^, Don Whitley Scientific, Bingley, UK) at 30 °C for seven days. The presence of a functional SRB community was confirmed by the characteristic production of H_2_S and black iron sulfide precipitates during the following experiments.

### 2.2. Pilot Tank System

The structure used in this work was a pilot tank system with a capacity of 8 L, which has structures to support AISI 1020 carbon steel coupons at dimensions of 10 × 10 mm, which provide a surface to form and analyze biofilm. To conduct this pilot-scale evaluation, the experiment was performed using two independent tanks designated as the “control” group and another two independent tanks designated as the “phage-treatment” group. SRB growth was conducted to allow the maximum anoxic environment. The tanks were compounds of 6.8 L of synthetic seawater (NaF 0.003 g/L, SrCl_2_·6H_2_O 0.02 g/L, H_3_BO_3_ 0.03 g/L, KBr 0.1 g/L, KCl 0.7 g/L, CaCl_2_·2H_2_O 1.113 g/L, Na_2_SO_4_ 4.0 g/L, MgCl_2_·6H_2_O 10.78 g/L, NaCl 23.5 g/L, Na_2_SiO_3_·9H_2_O 0.02 g/L, Na4EDTA 0.001 g/L, NaHCO_3_ 0.2 g/L, pH 8–8.1) filtered through a 0.45 μm membrane and heated until 45 °C, before adding 800 mL of a nutritive solution at a final concentration of 10X (yeast extract 10 g/L, 2.5 g/L glucose, 40 mL/L sodium lactate). Both solutions were exposed for 15 min to nitrogen gas. The respective mixed culture inoculum was transferred to the tanks using peristaltic pumps, and the treatment was applied in the same manner.

### 2.3. Bacteriophage Cocktail

The cocktail used in this study comprised four lytic bacteriophages: vB_EclM-UFV01 (UFV01), vB_EcoM-UFV09 (UFV09), vB_EcoM-UFV10 (UFV10), and vB_EcoM-UFV13 (UFV13). Detailed characteristics of each viral isolate, including host strains and taxonomic classifications, are summarized in Table 1. The phages were specifically selected for this formulation based on their proven capacity to significantly disrupt SRB biofilms.

Despite the >99% genetic identity between UFV09 and UFV10, both were maintained in the final 4-phage cocktail because they exhibited distinct biological behaviors during the preliminary analyses.

For the pre-inoculum, both bacterial species were growth in 300 mL of Luria Bertani (LB) medium, incubated for 24 h, 150 rpm, at 37 °C. The next day, the optical density (OD_600nm_) of the cultures was adjusted, and an inoculum of 600 mL with an OD_600nm_ of 0.1 was prepared. Phage propagation started when the culture reached logarithmic phase (OD_600nm_ 0.2–0.3), and a specific volume was used to obtain the final viral concentration of 10^7^ PFU/mL. Then, the bacterial culture and phage were incubated for 24 h, 150 rpm, at 37 °C. After the incubation time, the bacterial culture and viral suspension produced were transferred to 50 mL tubes and centrifuged at 8000× *g*, 4 °C, for 15 min, to obtain a cell-free viral suspension. Finally, the samples were filtered through a 0.22 μm pore membrane, with no further purification assays.

The serial dilution technique was used to determine viral titer. A total of 100 μL of each phage was mixed with 900 μL of SM buffer (5.8 g/L NaCl; 2.0 g MgSO_4_·7H_2_O; 50 mL Tris-HCl 1M; 5 mL gelatin 2%, pH 7.5). Then, 100 μL of each dilution was added to 900 μL of *E. coli* 30 and *E. cloacae* ATCC13047 (both in OD600 0.6), and the titration was performed using a double-agar method, incubated for 24 h at 37 °C. The four phages were mixed in an equal ratio (1:1:1:1) to obtain 80 mL of phage cocktail at a concentration of 109 PFU/mL, resulting in a final concentration of 10^7^ PFU/mL in the tanks. The phage cocktail was stored at 4 °C and only applied after the culture reached the H_2_S production peak.

### 2.4. Planktonic Cells Analysis

#### 2.4.1. Optical Density (OD) and H_2_S Titration

Using anaerobic tubes (Hungate), samples were taken daily in triplicate (10 mL) from the tanks to measure the optical density. The hydrogen sulfide concentration produced by the mixed culture in the tanks was monitored using an automatic titrator (Mettler Toledo^®^, Columbus, OH, USA) and AgNO_3_ as the titrant.

#### 2.4.2. Most Probable Number (MPN)

The most probable number (MPN) was carried out at three different time points: (1) just after the mixed culture inoculation in the tanks, (2) at the maximum production of H_2_S, and (3) on the last day of the experiment. An anaerobic and reduced saline solution (NaCl 3.5% m/v) was used for the serial dilution, ranging from 10^−1^ to 10^−7^. Next, 1 mL of each dilution was transferred to 9 mL of Postgate E medium (supplemented with FeSO_4_ 0.5 g/L). The MPN kits were incubated for 28 days at 30 °C, and SRB growth was indicated by a black precipitate in the tubes.

#### 2.4.3. ATP Quantification

The ATP quantification was performed using the LuminUltra^®^ ATP Testing Kit (Fredericton, NB, Canada) following the manufacturer’s instructions. The following formula shows the result in pgATP/mL:

cATP pg ATP/mL=RLUcATPRLUATP1×10,000(pg ATP)Sample volume (mL),

where RLU_cATP_ is the value found for the samples, and RLU_ATP1_ is the value obtained for the enzyme. As described before for MPN, for ATP, the concentration was measured at the same three specific times.

#### 2.4.4. DNA Extraction

To assess the impact of the phage cocktail on microbial community dynamics, samples for DNA extraction were collected at four defined time points: (1) day 1, following two hours of homogenization but prior to cocktail addition; (2) day 6, corresponding to the peak of H_2_S production—samples were taken after two sequential homogenization cycles, during which the phage cocktail was introduced between them; (3) day 19, representing the midpoint of the experiment; and (4) day 38, marking the end of the experimental period. These sampling points were consistently applied to both the control and treatment tanks.

The DNA extraction protocol was modified from Silva et al. [39]. Triplicate samples of 50 mL from each tank were centrifuged at 10,000× *g* g, 5 min, and the pellet was washed three times with SET buffer (20 mM Tris, 75 mM NaCl, 25 mM EDTA, pH 7.5). This procedure was repeated three times. Next, the pellets were resuspended in 1 mL of SET buffer, homogenized, and 50 μL of lysozyme (100 mg/mL solution) was added, incubated in a water bath at 37 °C for 30 min, and shaken every 10 min. Following this, 50 μL of proteinase K (10 mg/mL) and 200 μL of SDS 10% (final concentration in 2%) were added, and the solution was incubated in a water bath at 60 °C for 30 min, and shaken every 10 min again. After this, three freeze–thaw cycles (2 min in liquid nitrogen following 2 min at a water bath at 65 °C) were conducted. The same volume of buffered phenol (pH 8.0) was added to the solution, and after 2 min of homogenization, centrifuged at 10,000× *g* for 5 min.

The same volume of chloroform-isoamyl alcohol (24:1) was added to the recuperated superior phase and centrifuged at 10,000× *g* for 5 min. Then, NaCl 5 M (10% of total volume) and 2 volumes of cold ethanol were added to the solution, and a final centrifugation was performed at 10,000× *g* for 20 min. The pellets were washed with 70% ethanol and dried at room temperature. The dry pellet was resuspended in 50 μL of ultrapure water. The DNA was quantified using Qubit Fluorometric Quantification (ThermoFisher^®^, Waltham, MA, USA) and visualized in an agarose gel. The total DNA was sent for sequencing at Novogene (available online: https://en.novogene.com/ (accessed on 20 December 2021)) using the Illumina NovaSeq 6000 platform.

### 2.5. 16S rRNA Gene Amplicon Sequencing and Bioinformatic Analysis

Raw sequences were processed using DADA2 package v.128 [40]. Quality filtering and trimming were performed by removing the first 10 base pairs (bp) of all reads and truncating forward and reverse reads at 240 bp and 200 bp, respectively, retaining reads with a Phred quality score above 25. High-quality reads were then analyzed for the identification of the amplicon sequence variants (ASVs) present in the samples [41] and taxonomically classified using the Silva SSU database v.138 (available online: http://www.arb-silva.de (accessed on 3 August 2023)) [42].

To visualize dissimilarity between samples, principal coordinates analysis (PCoA) and dendrograms were conducted using the Bray–Curtis dissimilarity matrix to ordinate the data in two dimensions. Three different diversity indices, Shannon, richness, and Simpson, were used to measure diversity. Differences in ASV abundance between samples were tested using Kruskal–Wallis, and the resulting *p*-values were adjusted for multiple comparisons using the false discovery rate (FDR) method. All statistical analyses were executed in R (version 4.0) (R core team, 2021) with the support of the Vegan package [43], McToolsR (Available online: https://github.com/leffj/mctoolsr (accessed on 3 August 2023)), and Phyloseq [44].

A volcano plot was applied to demonstrate which ASVs were significantly enriched or depleted by treatments, using the tool MetaboAnalyst 5.0 [45], a *p*-value of 0.05 (with FDR correction), and an FC of +2 and –2 was used as the threshold.

### 2.6. Biofilm Assays

#### 2.6.1. ATP Concentration

The “Biofilm Collector” method available with the LuminUltra^®^ ATP Testing Kit (Fredericton, NB, Canada) was used to quantify the ATP concentration present on cells adhered to the coupons. These surfaces were immersed in an UltraLyse^®^ 7 Extraction Tube, vigorously agitated, and incubated for 5 min at room temperature. Subsequently, 1 mL of the samples was added to 9 mL of UltraLute^®^ and inverted three times. After Luminase^®^ verification, 100 μL of enzyme and 100 μL of sample dilution were mixed and then proceeded to the PhotonMaster^®^. The following formula was used to calculate the result:

tATP pg ATP/cm2=RLUtATP ×50,000RLUATP1× area (cm2)

#### 2.6.2. Scanning Electron Microscopy (SEM)

SEM was performed at the Microscopy and Microanalysis Center (Núcleo de Microscopia e Microanálise da Universidade Federal de Viçosa—NMM/UFV). The coupons were washed three times in PBS 1X (NaCl 8 g/L, KCl 0.2 g/L, Na_2_HPO_4_ 1.44 g/L, KH_2_PO_4_ 0.2 g/L, pH 7.4), and 4% paraformaldehyde (*v*/*v*) was used for fixation (26 h, at room temperature). Next, the coupons were washed again with 1× PBS and subjected to gradual ethanol dehydration (30%, 40%, 50%, 60%, 90%, and 100%) for 10 min per step, with the 100% stage repeated three times. For SEM visualization, coupons were dried at the critical point (Critical Point Dryer—CPD Bal-tec^®^ 030, Columbia Nano Initiative, New York, NY, USA), set on stubs, and covered with gold metallization (Sputter Coater Quorum^®^ Q150RS, Quorum Technologies, East Sussex, UK). Finally, these were observed using a Leo 1430VP (Zeiss, Oberkochen, Germany) scanning microscope. Different magnifications were used during image acquisition to capture both a broad overview of the general biofilm architecture and high-resolution microscopic details of the embedded microorganisms and their structural matrix.

#### 2.6.3. Quantification of Adhered Cells

Coupons were washed three times with anaerobic reduced saline. Cell detachment and quantification were performed by sonication in an ultrasonic bath containing fresh saline solution. Following three 1-min sonication cycles, 1 mL of the resulting sonicate was inoculated into the MPN kits, as described in Section 2.4.2. The kits were incubated at 30 °C for 28 days.

#### 2.6.4. Mass Loss and Corrosion Rate Measurements

The mass loss was determined by subtracting the initial mass of each coupon from its final mass measured at the end of the experiment. Corrosion products were chemically removed using a carbon-steel cleaning solution composed of 50% (*v*/*v*) hydrochloric acid and 3.5 g of hexamethylenetetramine, as specified in ISO 8407:2009. Coupons were exposed to this solution for 10–20 min, rinsed, dried, and subsequently weighed on an analytical balance. The corrosion rate was calculated according to ASTM G1–03 (American Society for Testing and Materials):

$$\frac{K\times W}{A\times T\times D}$$

where K is a constant 8.74 × 104 (mm/year), W is the mass loss (g), A is the area (cm^2^), T is the time to exposure (hours), and D is the material density (g/cm^3^).

#### 2.6.5. Optical Profilometry

Surface roughness of the biofilms was evaluated using a 3D optical profilometer (Contour GT-K, Bruker^®^, Billerica, MA, USA) at the Nanoscopy Laboratory, Federal University of Viçosa (SISNano/UFV). Coupons were washed three times with 1× PBS, oven-dried, and stored in a desiccator before analysis. The surface scans provided topographic maps and roughness parameters, including average roughness (Ra), maximum peak height (Rp), root mean square roughness (Rq), maximum valley depth (Rv), and the total height of peaks and valleys (Rt).

### 2.7. Statistical Analysis

Statistical analyses and data visualization were carried out using GraphPad Prism (version 8.4.3). Differences between the control and treatment groups (MPN, mass loss and corrosion rate, and ATP) were assessed using a Student’s t-test, with *p* < 0.05 considered statistically significant.

## 3. Results

### 3.1. H_2_S Production

**Figure 1:**
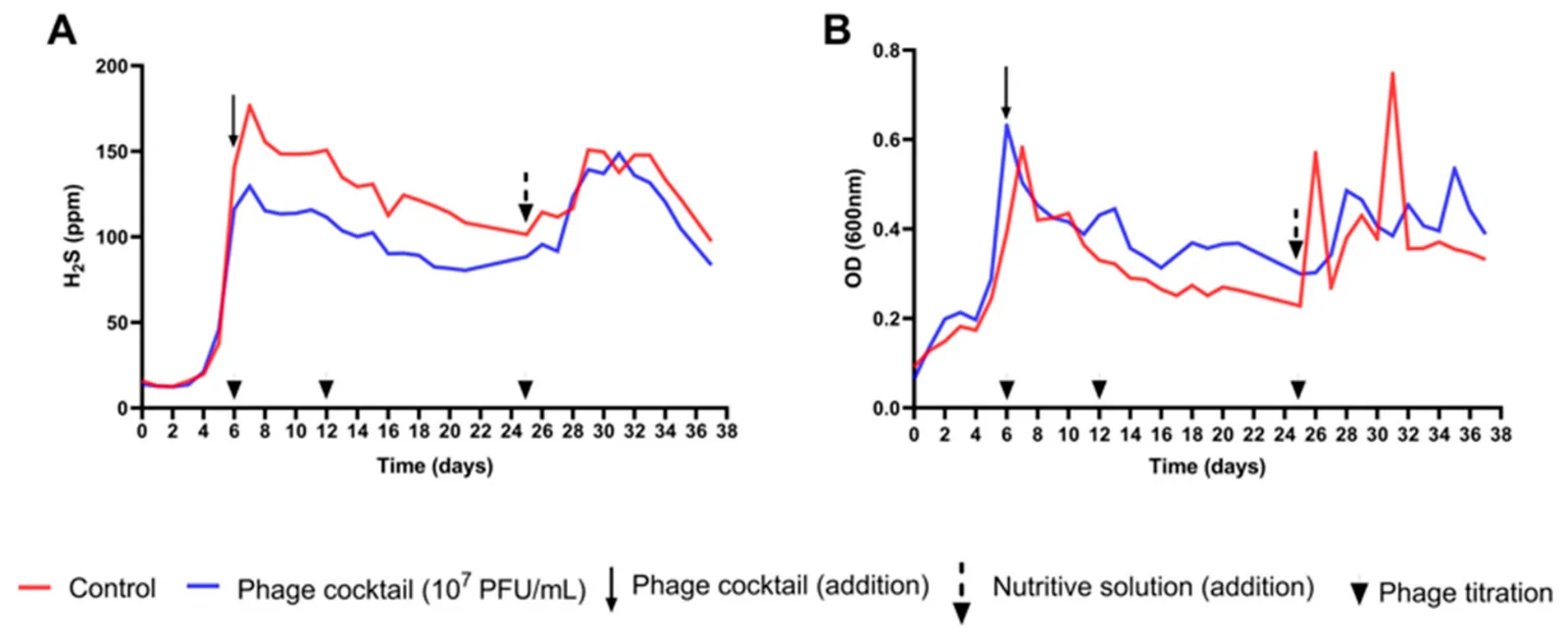
H_2_S production (**A**) and optical density (OD_600nm_) (**B**) of the AP culture over the 38-day experiment, comparing the control (red) with the phage cocktail treatment (blue). The solid arrow indicates the administration of the phage cocktail on day 6, which resulted in a pronounced decrease in H_2_S levels (**A**) and the disruption of planktonic growth (**B**) in the treated tanks. The dotted arrow represents the addition of a nutritive solution on day 25, demonstrating that the treated system failed to recover its initial activity despite nutrient replacement.

After six days of incubation, the mixed culture AP exhibited maximum H_2_S production in the experimental tanks, reaching 140.2 ppm in the control and 130.3 ppm in the treatment group (Figure 1A). At this point, the phage cocktail (10^7^ PFU/mL) was added to assess the subsequent response of the SRB community. The initial phage titer was 7.4 × 10^7^ PFU/mL. The first detectable reduction in H_2_S was observed on day 8, with a 26.1% reduction in the treatment, followed by 26.9% on day 17, and by day 38, the experiment concluded with a small difference between treatments.

After 6 days, the phage titer was 3.8 × 10^6^ PFU/mL, and on the day the nutrients were replenished, it was 6.9 × 10^5^ PFU/mL. The optical density at the 600 nm (OD_600nm_) profile (Figure 1B) indicated a decline in growth after phage introduction, consistent with the trend observed in the control. Upon supplementation with fresh medium, the control culture displayed two distinct growth peaks (0.568 on day 7 and 0.747 on day 14 after the new feed), while the treated culture reached its maximum OD_600nm_ of 0.537 on day 35, near the end of the experimental period.

### 3.2. MPN Analysis

**Figure 2:**
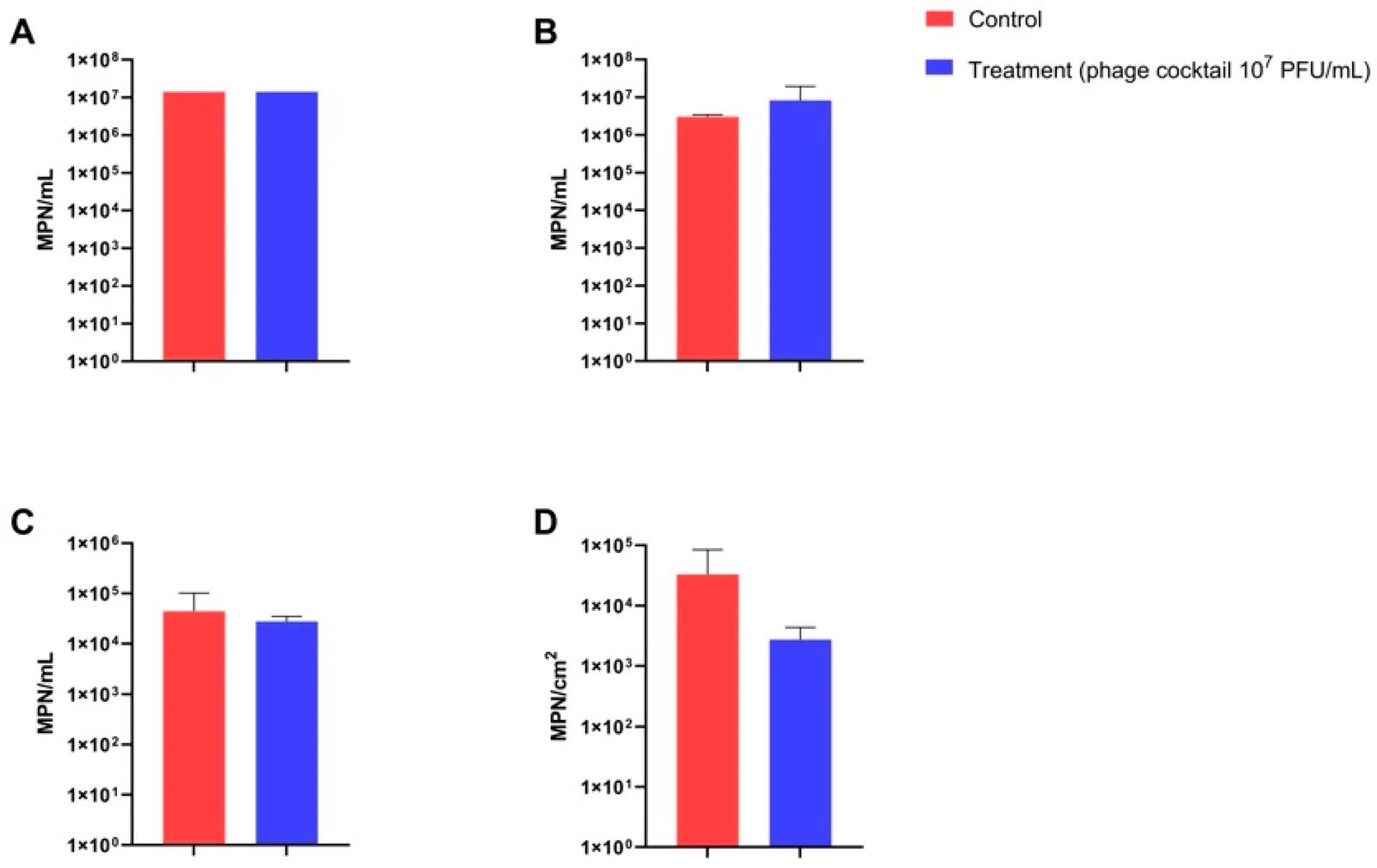
Most probable number (MPN) of the AP culture under phage cocktail treatment. Planktonic cell quantification (MPN/mL) at three different time points: (**A**) on the first day of the experiment; (**B**) on the 6th day; and (**C**) on the 21st day. (**D**) Quantification of adhered cells on coupons (MPN/cm^2^) after sonication at the end of the experiment, 38 days.

The cell concentration of the inoculum added to the tanks contained an estimated 1.4 × 10^7^ MPN/mL (Figure 2A). On day 6, coinciding with the peak of H_2_S production, the treatment and control tanks maintained close planktonic concentrations within the 10^6^–10^7^ MPN/mL range (Figure 2B), showing that the cocktail did not cause an immediate effect in the liquid phase. After 21 days (Figure 2C), both cultures exhibited a marked decline in cell numbers, with the treatment showing nearly a three-log reduction relative to the values found in the initial inoculum and at day 6. By day 38, MPN quantification of sessile cells (Figure 2D) was lower than that of planktonic cells, and the treatment tanks presented adhered cells numbering approximately one log below the control. Although there were numerical differences across the timeline, no statistically significant difference was observed.

### 3.3. Effect of the Phage Cocktail on ATP Concentration

**Figure 3:**
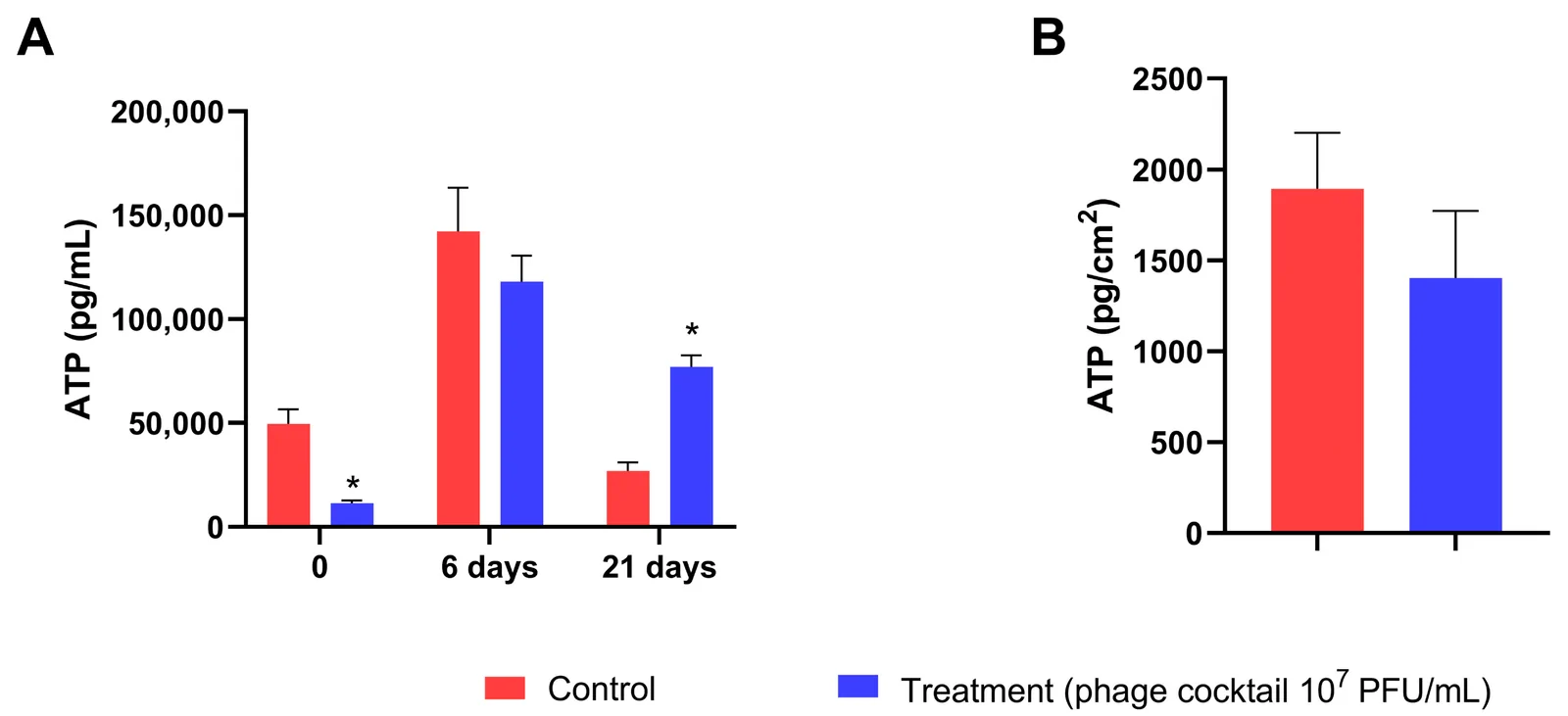
ATP concentration of the AP culture under phage cocktail treatment. (**A**) ATP concentration in planktonic cells measured at three time points: immediately after the AP inoculum (day 0), on the 6th day, and on the 21st day. (**B**) ATP concentration in adhered cells after sonication on the 38th day. (*) indicates a statistically significant difference compared to the untreated control (*p* < 0.05).

At the beginning of the experiment, the ATP concentration of planktonic cells differed markedly between tanks, with higher levels observed in the control compared to the pre-treatment tanks (Figure 3A). Six days later, before the addition of the phage cocktail, ATP concentrations in both populations converged. By the end of the experiment, the treatment tanks exhibited higher ATP levels than the control. After 38 days, the average biofilm ATP concentration measured on the coupons (Figure 3B) showed a 25% reduction in the treatment compared to the control, corresponding to 1500 pg ATP/cm^2^ and 2000 pg ATP/cm^2^, respectively.

### 3.4. Biofilm Structure by SEM

**Figure 4:**
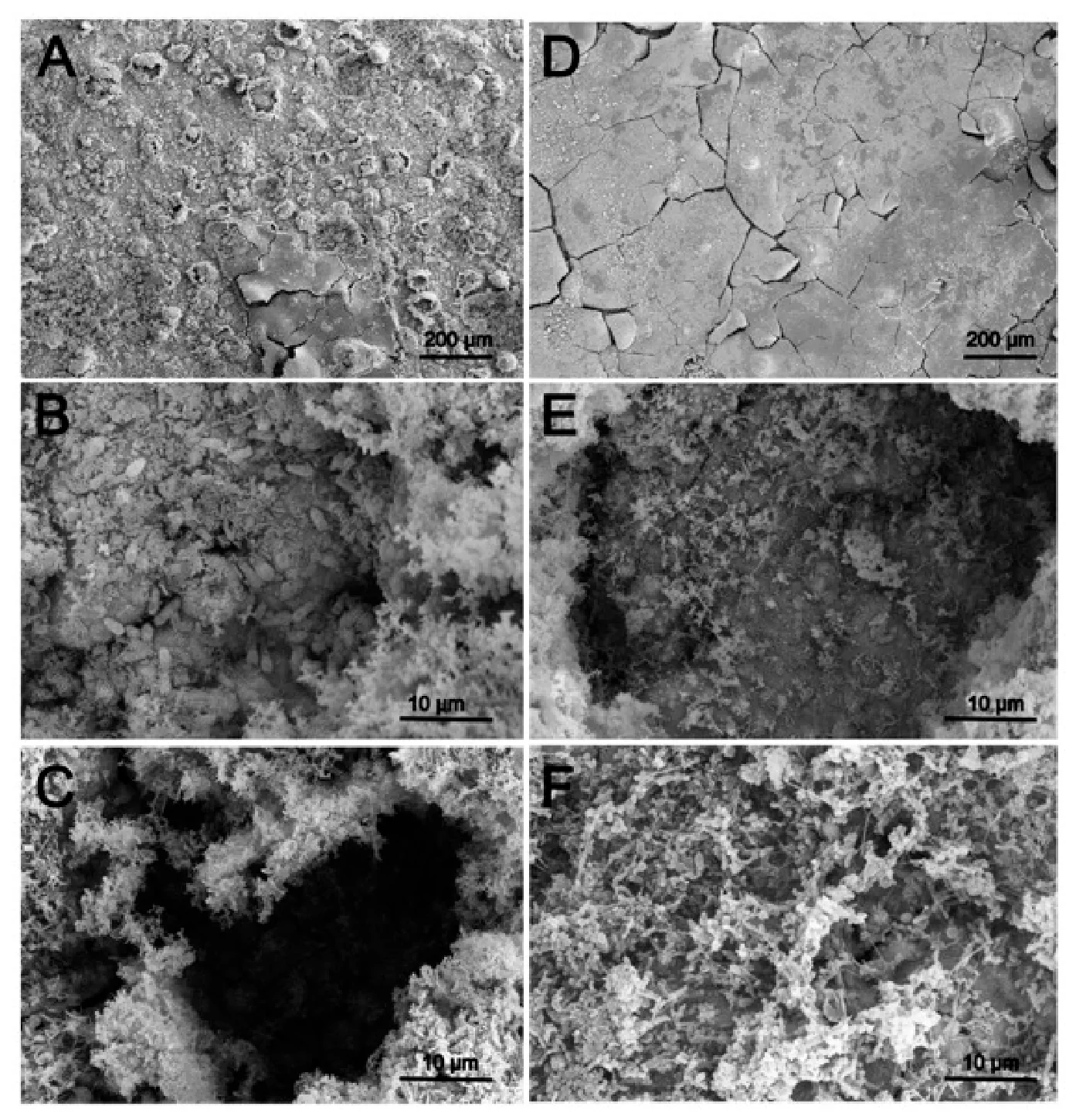
Scanning electron microscopy (SEM) images of the AP culture on AISI 1020 carbon steel coupons after 38 days. (**A**–**C**) Control tanks at 69×, 2620×, and 2380× magnifications, respectively. (**D**–**F**) Phage cocktail treatment tanks at 98×, 2620×, and 4140× magnifications, respectively. Reduced cellular adherence was observed on the treated surface (**E**) compared to the control (**B**), alongside the presence of cell filaments (**F**) following the phage treatment. Scale bars: 200 μm (**A**,**D**) and 10 µm (**B**,**C**,**E**,**F**).

The coupon surface of the control group exhibited pronounced degradation (Figure 4A), characterized by the presence of mature biofilms. In contrast, the phage-treated biofilm showed markedly reduced cell adhesion (Figure 4D) and limited EPS production. Cracks observed in the micrographs are attributed to alcohol-induced dehydration during sample preparation.

Mature biofilms displayed rod-shaped cells, typical of sulfate-reducing bacteria (SRB), and EPS deposits (Figure 4B,C). In the treated samples, cell density was visibly lower (Figure 4E), with a predominance of filamentous morphotypes (Figure 4F), possibly reflecting a cellular response to phage activity or differences in bacterial species content.

### 3.5. Mass Loss and Corrosion Rate

**Figure 5:**
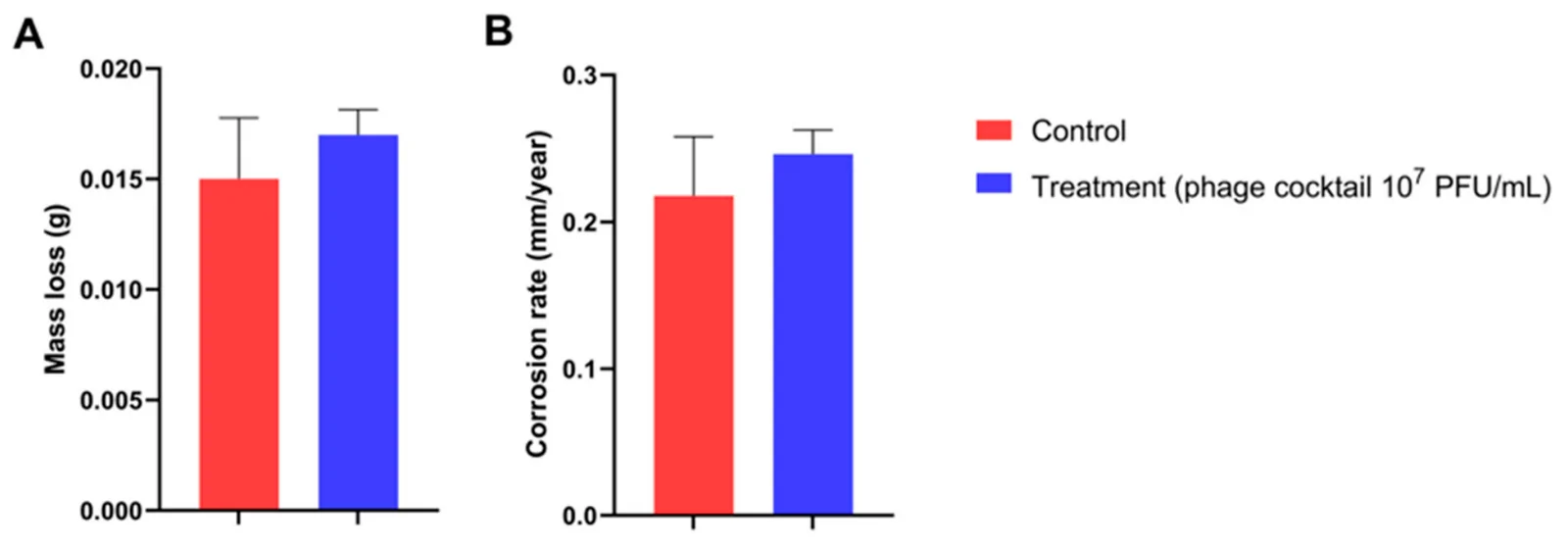
Mass loss (**A**) and corrosion rate (**B**) of the AISI 1020 carbon steel coupons after exposure to the AP culture over 38 days, comparing the untreated control with the phage cocktail treatment (10^7^ PFU/mL).

Both tanks exhibited comparable parameter values, with no statistically significant differences in mass loss (Figure 5A) and corrosion rate (Figure 5B). The corrosion rate was 0.2176 mm/year for the control coupons and 0.2467 mm/year for the phage-treated samples.

### 3.6. Surface Roughness

The quantitative roughness analysis is presented in Table 2. Surface profilometry revealed that despite similar average roughness (Ra) values, the vertical amplitude of the surface was markedly reduced by the phage cocktail. The total height of peaks and valleys (Rt) decreased from 156.95 in the control to 130.57 in the treated coupons, representing a 16.8% reduction in overall surface irregularity.

**Figure 6:**
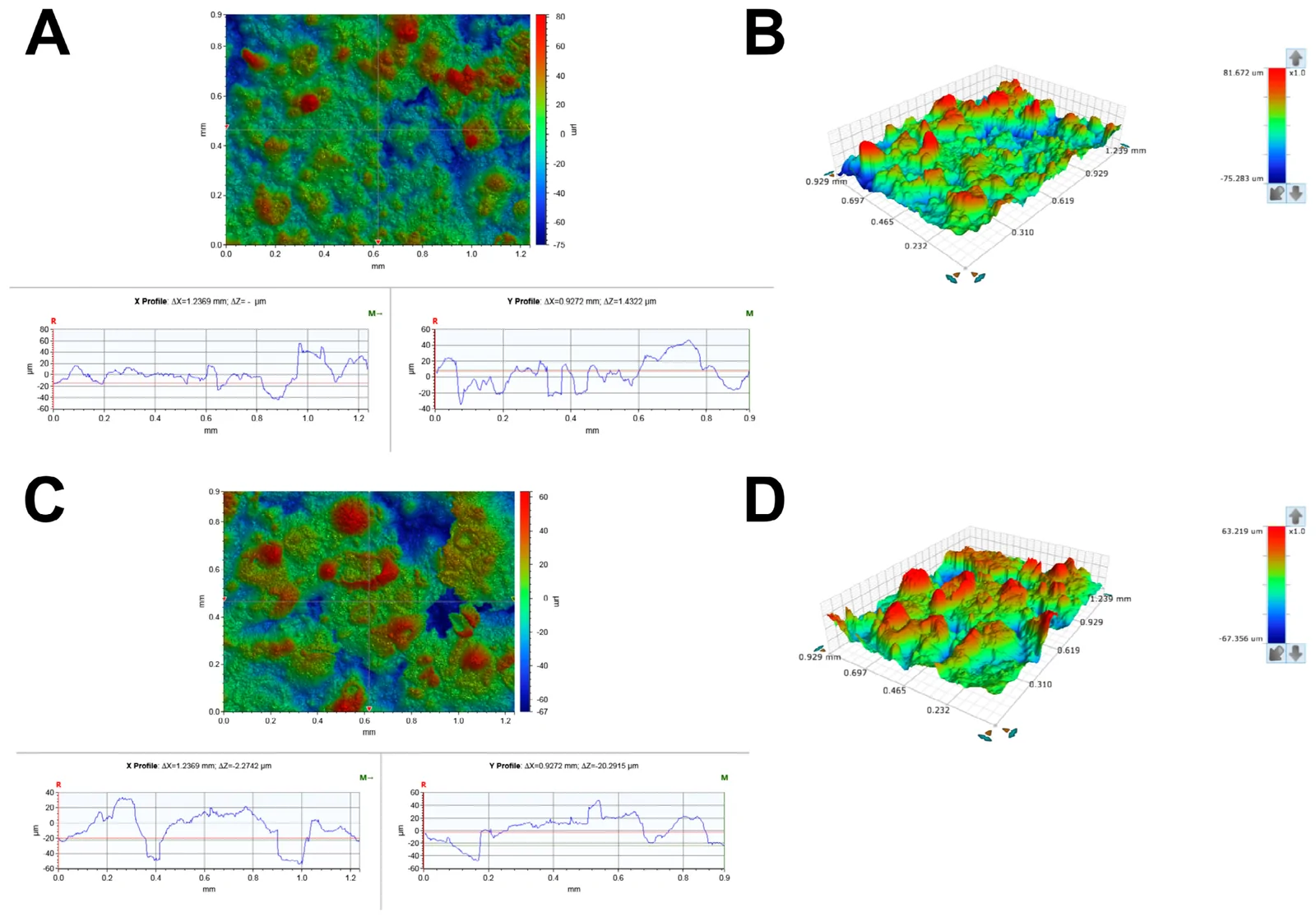
Optical profilometry of the biofilm formed by AP culture on the AISI 1020 coupons after 38 days of experiment. (**A**) Two-dimensional (2D) surface topography map and corresponding extractable X and Y linear profiles for the control tanks, showing pronounced vertical valleys. (**B**) Three-dimensional (3D) surface reconstruction of the control coupon highlighting intense, localized pitting corrosion. (**C**) 2D surface topography map and corresponding X and Y linear profiles for the phage cocktail treatment tanks. (**D**) 3D surface reconstruction of the treated coupon, demonstrating a reduction in pit depth and peak amplitudes.

This reduction in vertical scale was visually confirmed by comparing the control coupon (Figure 6A) with the treated coupon (Figure 6C). A distinct difference in the spatial distribution of surface irregularities was also observed: in the control, surface valleys were more numerous and pronounced along the *Y*-axis (Figure 6A), whereas the treated surface exhibited a higher concentration of valleys along the *X*-axis (Figure 6C). Furthermore, the decrease in Rv values in the treated group demonstrates the ability of the phage cocktail to limit the depth of corrosion pits. Three-dimensional (3D) reconstructions further indicated a predominance of peaks (Rp) in both tanks; however, these peaks were significantly smaller in the cocktail-treated coupons (63.22 μm) compared to the control (81.67 μm) (Figure 6B and Figure 6D, respectively).

### 3.7. Diversity Analysis

**Figure 7:**
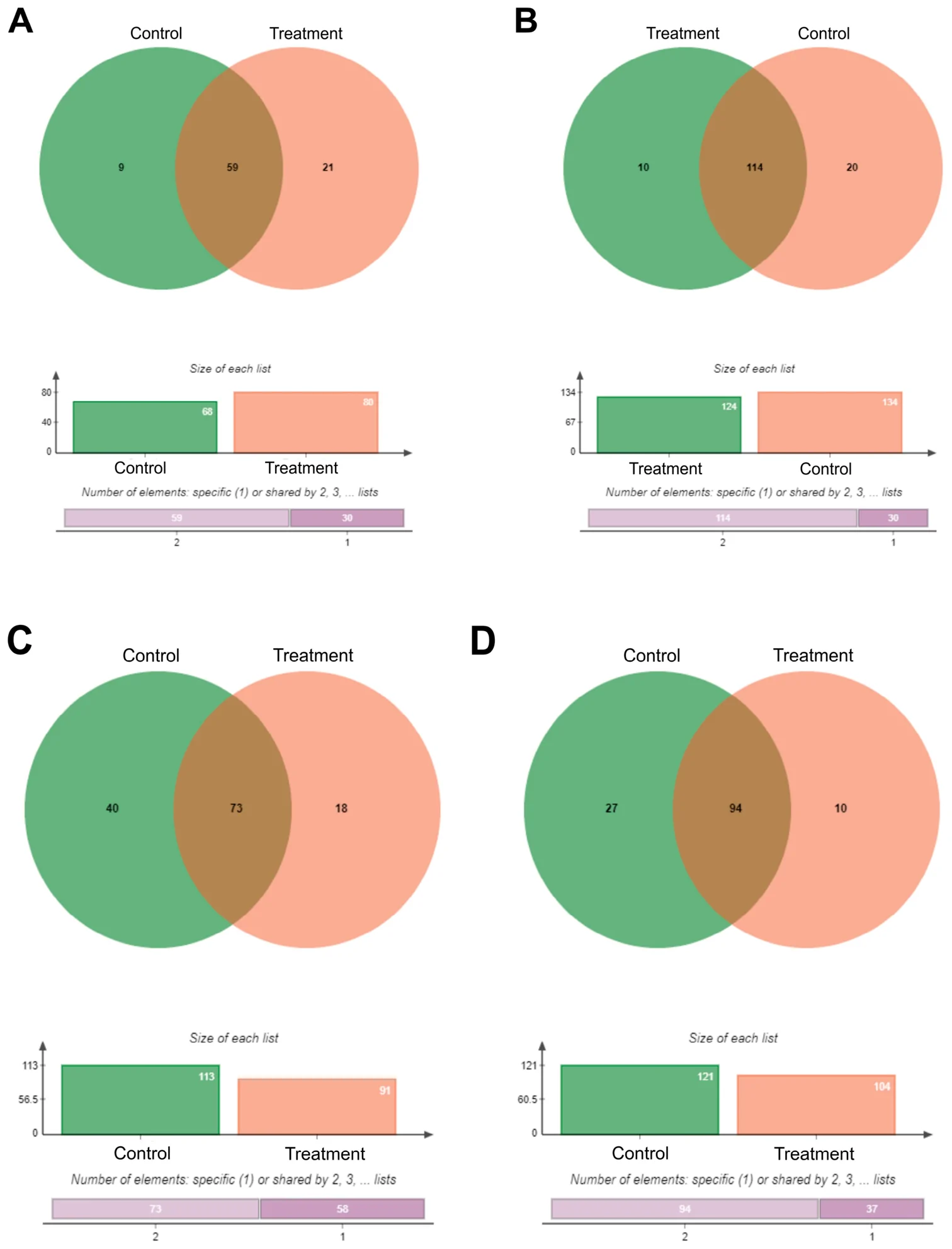
Venn diagram of microorganism genera after metagenomics analysis according to the time point of the experiment. (**A**) time point 1; (**B**) time point 2; (**C**) time point 3; (**D**) time point 4.

The Venn diagram illustrates the shared and unique bacterial genera between the control and treatment tanks over time (Figure 7). At time point 1 (Figure 7A), a total of 59 genera were common to both tanks. This number increased to 114 at time point 2 (Figure 7B), while the number of treatment-specific genera remained relatively constant (1 and 2), indicating an overall enrichment of bacterial diversity in both tanks without marked differences between them. At time point 3 (Figure 7C), the number of shared genera decreased to 73. Concurrently, the control tanks exhibited a twofold increase in the number of genera, while the treatment tanks retained a similar richness (Figure 7C). At the end of the experiment, shared genera rose again to 94 (Figure 7D).

The most pronounced divergence in the composition of unique genera was observed at time point 3 (Figure 7C). At this stage, the treatment tanks contained only 18 unique genera, the lowest count compared to the control, which presented 40 unique genera. Before phage addition, the control tanks exhibited fewer unique genera than the treatment tanks. This pattern reversed after phage application, with the treatment tanks showing a reduced number of unique genera compared to the control.

**Figure 8:**
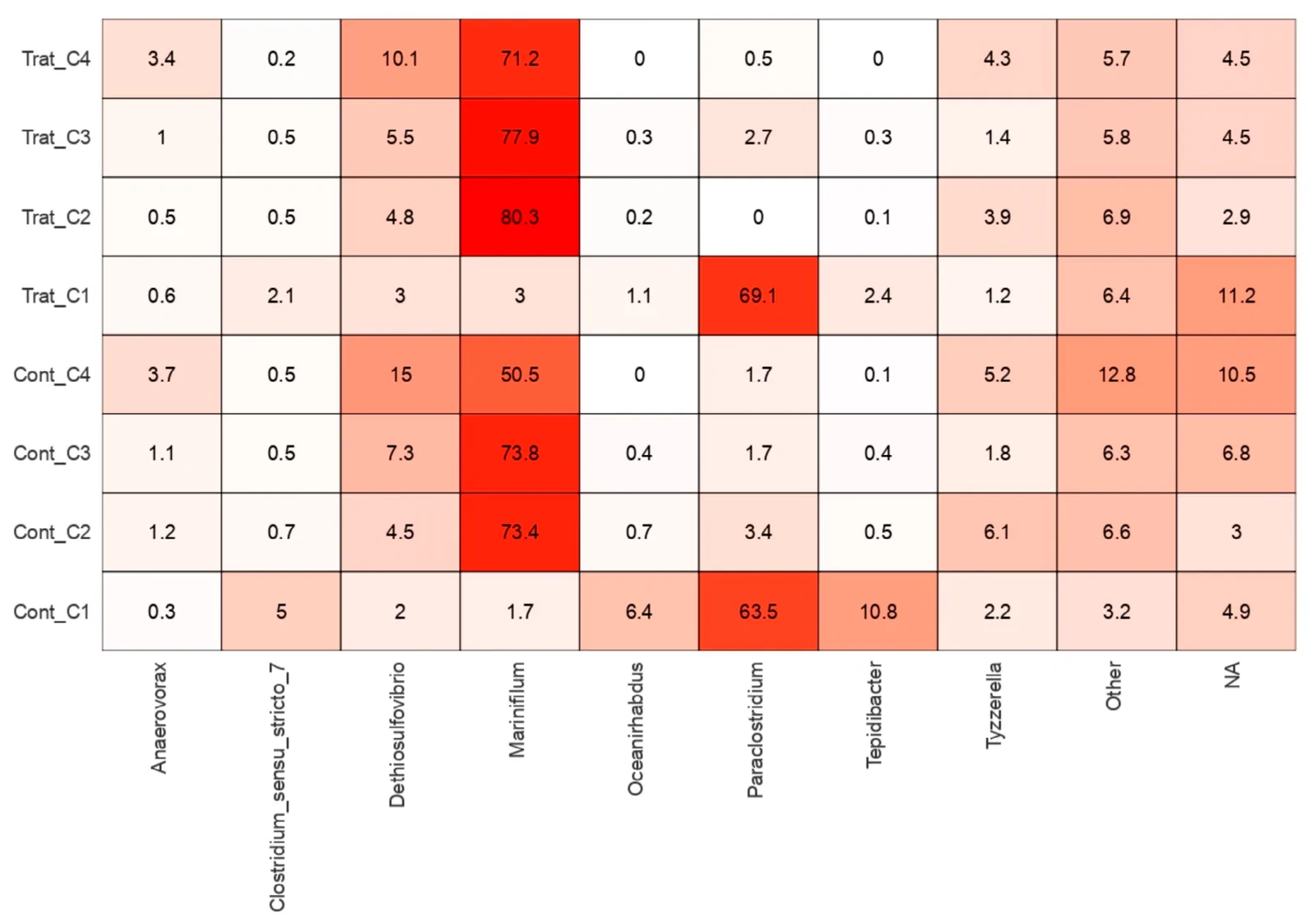
Heat map of the bacterial genera content during the AP culture experiment. On the left side, control samples are denoted as “Cont”, and treatment as “Trat”. Relative abundance is indicated by a color scale from white (low) to dark red (high), with the corresponding percentage values shown inside the cells. The highest values are emphasized in bold. The *Marinifilum* was the most abundant at all time points.

The heat map analysis (Figure 8) indicated a consistent prevalence of the genus *Marinifilum* at three out of the four time points for both the control and treatment, except at time point 1, where *Paraclostridium* predominated. After phage inoculum (time points 3 and 4), it was possible to observe the reduction in the *Dethiosulfovibrio* genus in the treatment tanks.

**Figure 9:**
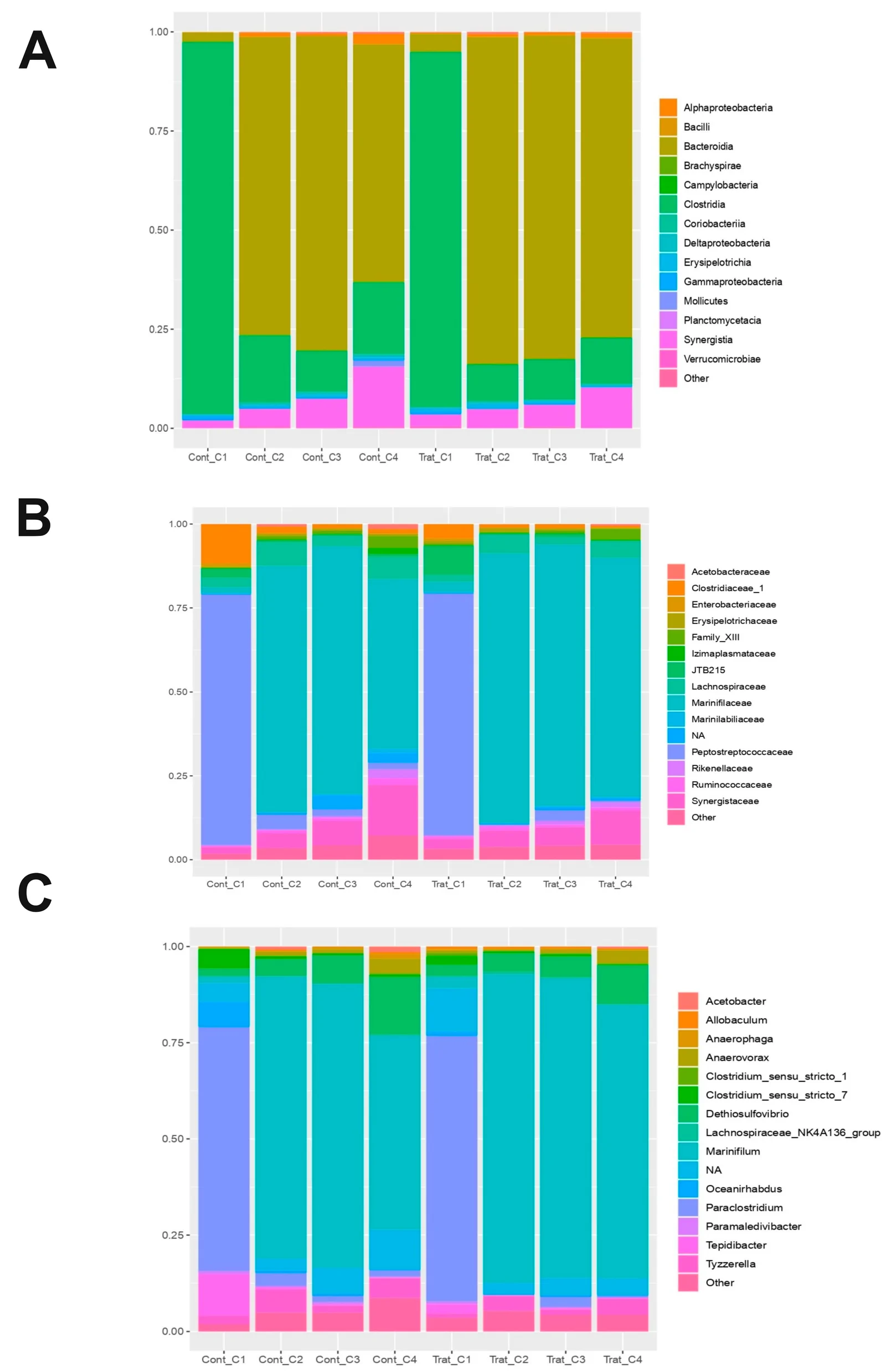
Taxonomic composition and relative bacterial abundance (from 0.00 to 1.00) during the AP culture experiment. (**A**) Class-level distribution. At time point 1, in both experiments, Clostridia was the most abundant class, followed by Bacteroidia. (**B**) Family-level distribution. Peptostreptococcaceae is highlighted at time point 1 for both the control and treatment, followed by Marinifilaceae. (**C**) Genus-level distribution. *Paraclostridium* was the main genus at time point 1 for both the control and treatment, followed by *Marinifilum*.

Taxonomic analysis at the class, family, and genus levels revealed distinct successional patterns across both tanks (Figure 9). Initially, the community was dominated by the class Clostridia (Figure 9A), the family Peptostreptococcaceae (Figure 9B), and the genus *Paraclostridium* (Figure 9C). In the next time points, Bacteroidia became the prevailing class, while Gammaproteobacteria increased in both tanks, although slowly in the treated one. At the family level, the early dominance of Peptostreptococcaceae and Clostridiaceae was succeeded by Marinifilaceae, Marinilabiliaceae, and Synergistaceae. By the final time point, the control tanks exhibited notably higher proportions of Synergistaceae and Peptostreptococcaceae compared to the treatment. At the genus level, *Marinifilum* became dominant from day 6 onwards; however, by the end of the study, *Dethiosulfovibrio* was significantly more abundant in the control than in the treated tank.

**Figure 10:**
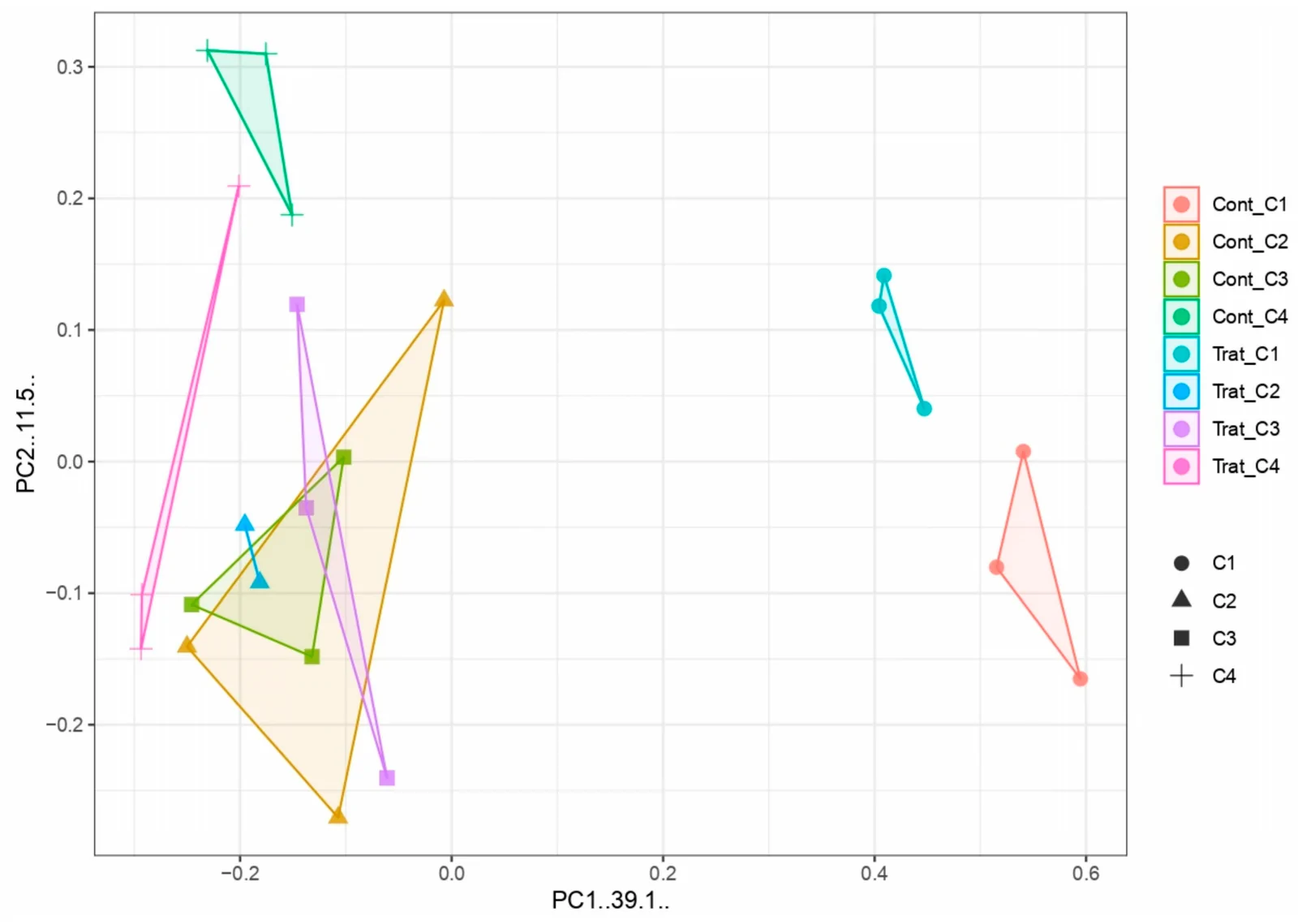
Principal coordinates analysis (PCoA) of bacterial community composition. Proximity between points indicates higher compositional similarity, whereas greater spatial distances reflect increasing dissimilarity. Samples from time point 1 exhibit marked separation from subsequent time points, reflecting distinct community structures across sampling intervals.

Principal coordinate analysis (PCoA) was used to assess similarities and differences in microbial community composition (Figure 10). PCoA1 and PCoA2 accounted for 39.1% and 11.5% of total variance, respectively. At time point 1, both the control and treatment samples were clearly separated from later time points, indicating distinct initial community structures. At time point 2, the control group displayed the highest intra-group variability. At time point 3, both groups exhibited some dispersion, particularly the treatment, which contained one divergent sample. By time point 4, microbial communities in both groups appeared to undergo structural reorganization. Overall, the control exhibited a more cohesive and stable community over time, whereas the treatment showed greater variability, likely reflecting the influence of the phage cocktail.

**Figure 11:**
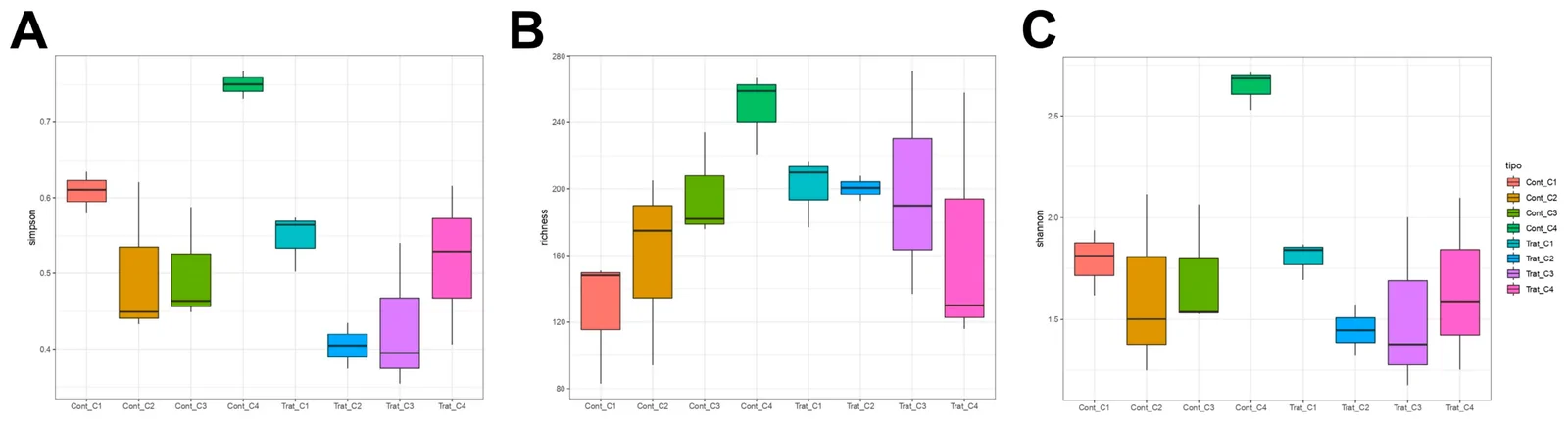
Box plots for the Simpson (**A**), richness (**B**), and Shannon (**C**) indices of the AP culture during the 38 days of the experiment. On the right side, control samples are denoted as “Cont”, and treatment as “Trat”. “Cont_C4”, corresponding to time point 4, was the highest. Boxes represent the interquartile range; the horizontal line represents the median; whiskers correspond to the lowest and highest values.

Alpha diversity was assessed using Simpson, richness, and Shannon indices. The final control sample (“Cont_C4”) showed the highest values across all three metrics. The Simpson index (Figure 11A) showed similar behavior at the beginning and at time points 2 and 3, but presented a remarkable difference at time point 4. After initially falling in both tanks, the value found in the control tanks exceeded the initial value by 1.5, while the treatment tanks simply returned to the initial value. Richness (Figure 11B) exhibited two distinct patterns: in the control tanks, it increased continuously, whereas in the treatment tanks it declined over time, reaching its lowest value (similar to point 1 in the control) at the end of the experimental period. The Shannon index (Figure 11C) reached its highest value in the control tanks at time point 4, exhibiting a pattern similar to that observed for the Simpson index.

**Figure 12:**
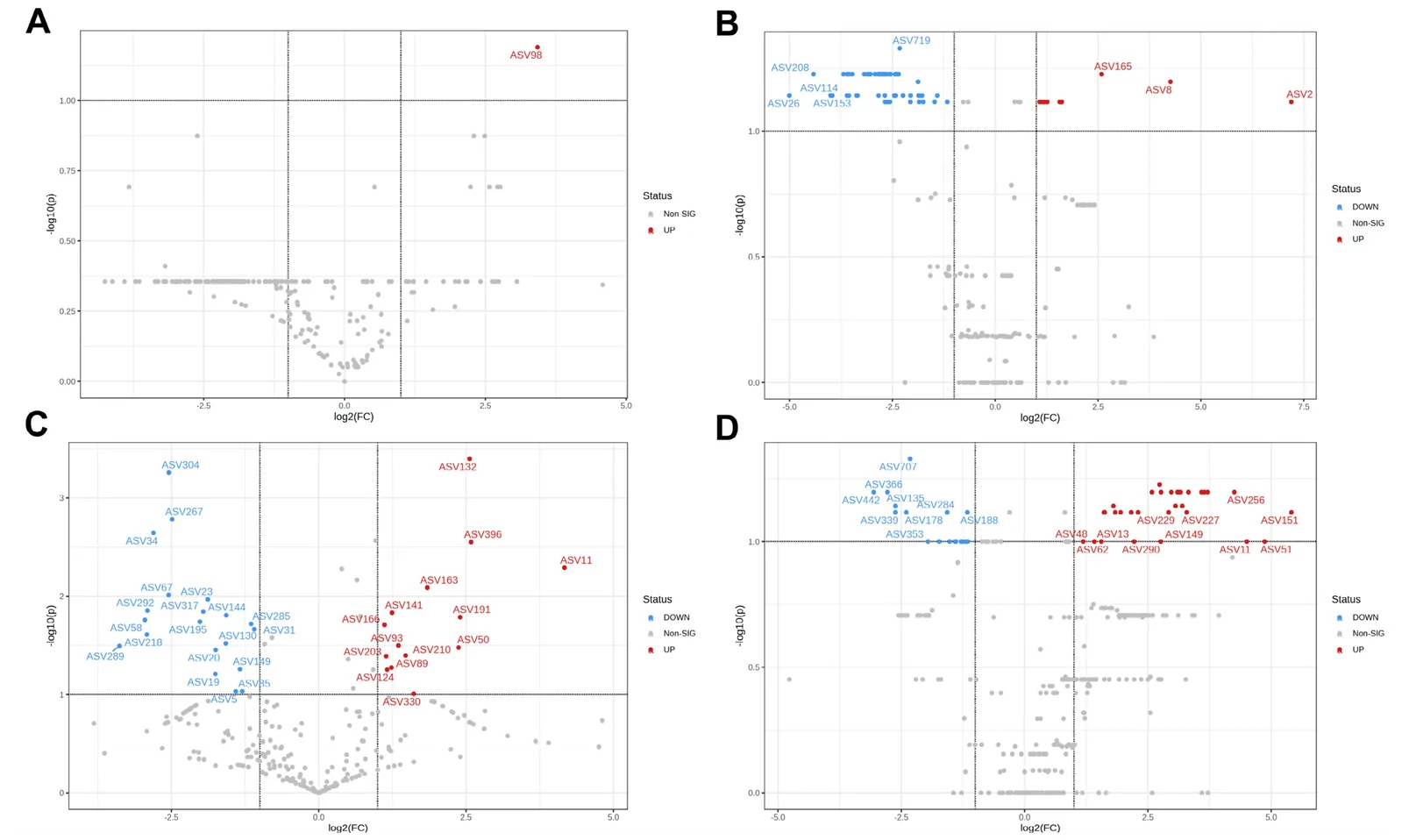
Volcano plots associated with the ASVs during time points 1 (**A**), 2 (**B**), 3 (**C**), and 4 (**D**). The depleted (“down”) ASVs are indicated in blue, and the enriched (“up”) in red. The Y axis determines the significance.

Differential 1 was very similar, with only one ASV98 differentially present (Figure 12A). At time point 2, two hours after phage inoculum, a few ASVs were enriched, and more were significantly depleted (Figure 12B). Greater variation occurred at later stages: at time point 3 (Figure 12C), 14 ASVs were enriched and 20 depleted, while at time point 4 (Figure 12D), 11 ASVs were enriched and 9 depleted.

**Figure 13:**
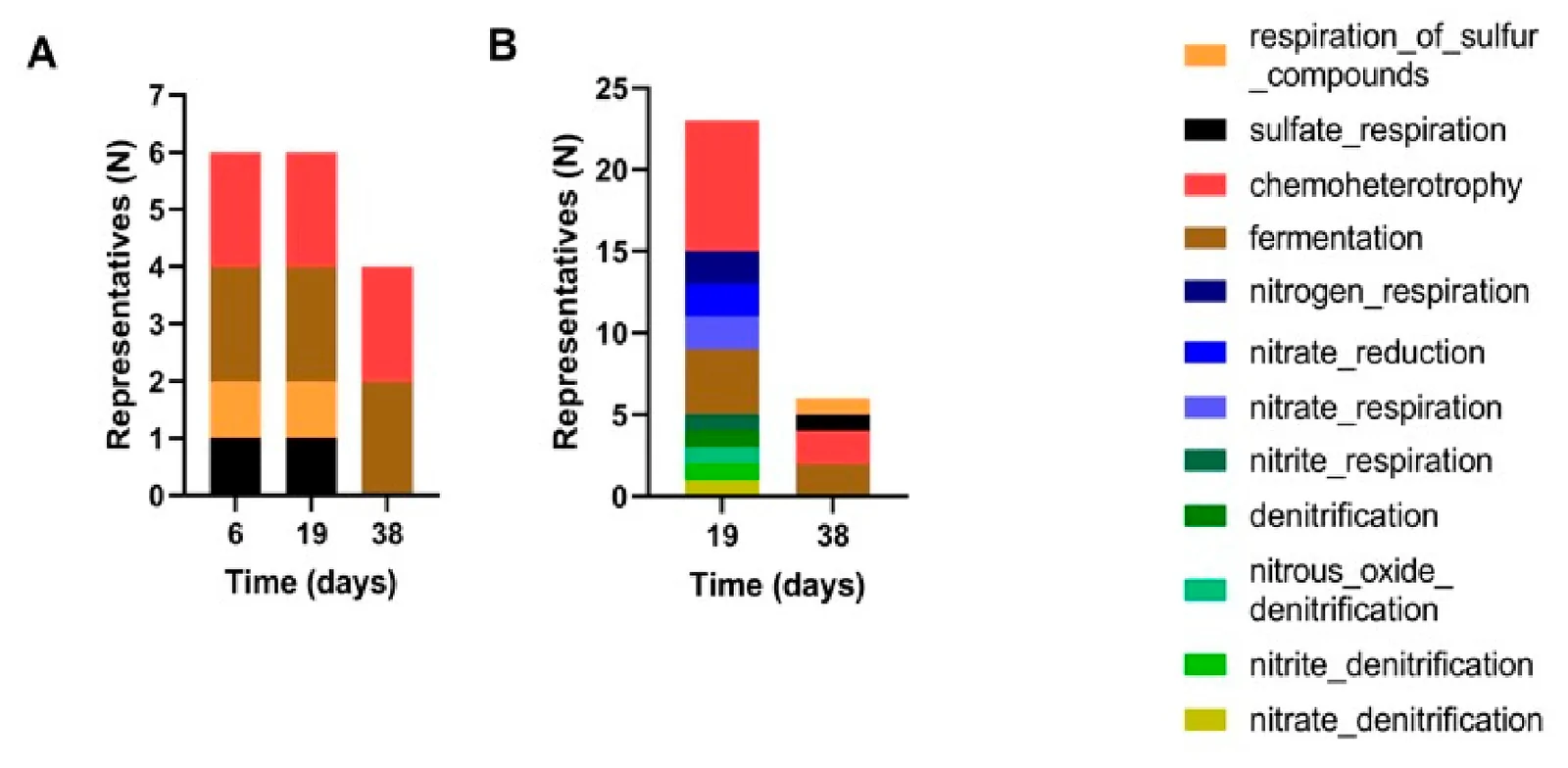
Distribution of microbial functional groups in the treatment tanks over time. (**A**) Functional groups were enriched at 6, 19, and 38 days. (**B**) Functional groups were depleted at 19 and 38 days.

Based on ASVs differentially present in the treated tanks, predictive functional profiling was performed across all four time points (Figure 13). These functional categories represent in silico predictions inferred from taxonomic 16S rRNA gene data and do not represent direct metabolic activities. Enriched ASVs (Figure 13A) at time points 2 and 3 were mainly linked to the potential functional groups “sulfate respiration”, “respiration of sulfur compounds”, “fermentation”, and “chemoheterotrophy”. By time point 4, only “fermentation” and “chemoheterotrophy” remained increased. ASVs depleted by the phage cocktail (Figure 13B) at time point 3 were associated with ten potential functional categories, including “nitrate denitrification”, “nitrite denitrification”, “nitrous oxide denitrification”, “denitrification”, “nitrite respiration”, “fermentation”, “nitrate respiration”, “nitrate reduction”, “nitrogen respiration”, and “chemoheterotrophy”. By the final sampling point (4), the potential functional groups associated with downregulated ASVs included “fermentation”, “chemoheterotrophy”, “sulfate respiration”, and “respiration of sulfur compounds”. No functional associations were observed for depleted ASVs at time points 1 and 2.

## 4. Discussion

In the present study, we applied a cocktail of non-specific bacteriophages against a mixed SRB culture, here referred to as AP, within a pilot-scale tank designed to mimic storage tanks used in the gas industry. This phage-based approach has emerged as a promising biocontrol strategy [46], offering specificity toward both narrow and broad bacterial host ranges. Most conventional methodologies used to evaluate phage activity rely on the double-agar method for isolation and titration [47], or static microplate assays to assess biofilm [48]. While these in vitro methods serve as a fundamental baseline, they present inherent methodological limitations for biofilm evaluation, like the frequent underestimation of viable cells due to structural aggregation and false biomass interpretations caused by residual matrix components [49]. In this context, the pilot-scale system represents a biotechnological innovation, demonstrating the applicability of phages against isolated strains and a complex, multi-species biofilm under realistic dynamic conditions.

A reduction in H_2_S concentration was observed after phage addition, but OD values in the treatment tanks exceeded those in the control. Nutrient re-addiction occurred on day 25; however, ATP levels in the treatment tanks were already substantially higher by day 21, consistent with the OD trends. The addition of nutrients reestablished microbial growth, particularly in the control tanks, which showed pronounced OD peaks, while the H_2_S levels remained similar between tanks. Although phages may have modulated the community structure, nutrient availability was sufficient to support microbial regrowth [50,51].

Previous studies have documented the efficacy of virulent phages in reducing bacterial growth [52,53,54,55]. Nevertheless, isolating SRB-specific phages is challenging due to the anaerobic conditions required for host cultivation and the high number of different species in this group. A recent study by Zhang et al. [56] described a phage infecting *Desulfovibrio vulgaris* (*Nitratidesulfovibrio vulgaris*) that not only exhibits lytic activity but also produces enzymes that degrade biofilms. The cocktail used in this study contains phages known to encode enzymes with biotechnological potential [57,58] and are capable of biofilm disruption. The application of phage-encoded depolymerases has demonstrated highly promising results in biofilm eradication in various scenes [59,60,61], as these enzymes efficiently degrade the EPS that stabilize the matrix. Therefore, the enzymatic degradation of the EPS matrix circumvents the need for SRB-specific phages, making this cocktail an advantageous strategy for disrupting mixed microbial biofilms. Finally, this degradation alters the taxonomic diversity and compromises the structural integrity of the entire biocorrosion system.

SEM analysis (Figure 4) revealed the deposition of exopolysaccharide-like material on the coupon surfaces. Control coupons exhibited mature biofilm formation, whereas those from the treatment tanks showed reduced cell adhesion or possible phage-induced disruption of the biofilm matrix. Since biofilm structures accelerating the corrosion process have been reported [62], biofilm disassembly is correlated with a reduction in both H_2_S and ATP concentrations, as assessed at the end of the experiment on the coupons (Figure 3B). Rod-shaped and coccobacillus-shaped SRB were prevalent on the control coupons (Figure 4C), whereas the treatment coupons showed predominantly filamentous structures (Figure 4F). Filamentous morphotypes are associated with cell-to-cell communication and facilitate contact with adhered surfaces [63,64], but also indicate a stress-induced inhibition of cell division [65,66].

These morphological changes are supported by the profilometry results (Figure 6; Table 2). The higher Rp values in the control reflect the accumulation of a more voluminous and mature biofilm, as observed in the SEM images, and the deeper Rv metric confirms that the absence of treatment allowed for more aggressive localized pitting. Pitting morphology is an indicative signal of MIC and has been evaluated in industrial materials such as carbon steel [67] and X80 pipeline steel [62]. Although Ra and Rq were similar between tanks (probably due to the presence of residual biofilm debris), the reduction in the Rt value in the treated tank demonstrates the effectiveness of the phage cocktail.

Recent studies using dual anaerobic reactor models [68,69] have highlighted the limitations of conventional biocides, such as glutaraldehyde, which often fail to completely eradicate mixed-species biofilms and can lead to the enrichment of stress-tolerant taxa. Even after biocide dosing, the study showed that biofilms persisted and induced significant localized corrosion, with a 3.7-fold increase in pit area compared to abiotic conditions. In a complementary manner, our results demonstrate that the phage cocktail suppressed planktonic community diversity in the bulk liquid while simultaneously disrupting the biofilm’s structural integrity, as evidenced by the reduction in Rv and Rt values. This suggests that phage-based formulations may offer a promising and sustainable complementary tool, potentially bypassing some of the chemical tolerance and diffusion barriers often associated with traditional treatments.

Microbial communities respond rapidly to environmental fluctuations, such as nutrient availability, oxygen levels, and biological or chemical stressors, and the presence of phages can influence, both directly and indirectly, which populations become established [70]. This dynamic behavior was clearly reflected in our metagenomic results. The Venn diagram (Figure 7) revealed the highest number of shared genera at time point 2; however, by the end of the experiment, a divergence was noted, with the control tanks containing 121 genera compared to 104 in the treatment tanks. These genera, visualized via heatmap (Figure 8) and bar plots (Figure 9C), showed a predominance of *Marinifilum*, *Paraclostridium*, and *Dethiosulfovibrio*, which are typical of marine substrates. These taxa represent distinct physiological groups: *Marinifilum* comprises Gram-negative, facultative anaerobic species [71], while *Paraclostridium* includes Gram-positive, anaerobic motile representatives such as *P. bifermentans* [72]. *Dethiosulfovibrio*, a genus of strictly anaerobic, Gram-negative bacteria associated with thiosulfate and elemental sulfur reduction [62] were detected at lower abundance but increased steadily over time in the control group. Given its role in converting sulfite to sulfide [73], its suppressed presence in the treated tank aligns with the lower sulfide concentrations observed, further supporting the efficacy of the phage treatment.

While the mixed-culture inoculum used in this study has previously been characterized [74], our 8-L pilot system encompassed a significantly broader range of microorganisms (Figure 9). This increased diversity likely stems from the non-sealed nature of the pilot system compared to the penicillin flasks used previously, allowing for more complex and real ecological succession. Notably, classes within the Proteobacteria (Alphaproteobacteria, Deltaproteobacteria, and Gammaproteobacteria), along with other sulfur-reducing organisms, were less abundant in the treated tanks, which supports the hypothesis of phage-mediated community modulation.

Throughout the 38-day experiment, both tanks developed distinct microbial communities, reflecting different ecological trajectories. The significant surge in alpha diversity metrics in the control tanks reflects the predictable trajectory of primary microbial succession toward a climax community [75], where the established environment created diverse ecological niches for secondary fermentative and chemoheterotrophic taxa (Figure 13). Bacterial communities transition from an early stage, marked by stochastic colonization by opportunistic pioneers, to a late stage, where taxonomic diversity is generally higher due to increased habitat heterogeneity and resource diversity [76]. This ecological maturation aligns with the decline in H_2_S concentrations and OD values at the end of the experiment, which is consistent with the slow growth rate typical of mature communities, where stability and intense interspecific competition replace the initial rapid growth phase. While the control group apparently reached this climax stage and metabolic stability, the maintenance of reduced diversity in the treated group (Trat_C2 to Trat_C4) may be occurring through the influence of the phage cocktail, which interrupted this natural succession in the planktonic phase. This continuous ecological disturbance in the liquid phase, combined with the possible EPS matrix disruption and keeping the system under constant stress (as seen in Figure 4), probably restricted the recruitment of species for biofilm stabilization, thereby preventing the organization of multispecies community on the metal and mitigating localized corrosive attack [77].

The temporal patterns observed in Figure 13 underscore shifts in metabolic groups that were either favored or suppressed. Among the enriched groups, fermentative and chemoheterotrophic organisms were prominent across all time points, likely reflecting anaerobic conditions combined with available carbon sources, consistent with the OD increase. H_2_S production peaked on day 6 (Figure 1A), corresponding to the enrichment of sulfate-respiring and sulfur-compound-respiring groups. However, the nutrient solution added on day 25 contained glucose and sodium lactate but no sulfate, leading to two interpretations: the phage cocktail may have suppressed enrichment of these groups, or the absence of sulfate prevented SRB proliferation. A previous study by our group showed that one of the phages in the cocktail, UFV13, requires a bacterial consortium (*Oceanidesulfovibrio marinus* and *Oceanotoga teriensis*) to establish infection and effectively reduce ATP metabolism, H_2_S production, and OD [78].

Figure 13B shows heterogeneous depletion patterns, particularly among fermentative and chemoheterotrophic organisms. This suggests a modulatory effect of the phage cocktail, consistent with evidence that phages shape natural communities through both direct lysis and indirect ecological effects that favor non-susceptible hosts [79,80]. Thus, while some members of a functional group were enriched, others were depleted. Notably, denitrifying bacteria were depleted on day 19. Although these organisms are often intentionally stimulated in industrial control strategies (e.g., nitrate injection to outcompete SRB), excessive denitrifier accumulation can promote MIC [81]. This behavior may be related to nutrient competition and the indirect effect of phages on the community.

By day 38, SRB depletion was consistent with the absence of SRB-enriched profiles. The alternating enrichment and depletion of fermentative and chemoheterotrophic organisms further support the hypothesis of microbial modulation by phages. OD data indicated microbial regrowth even after phage application, and functional analysis showed no depleted groups before treatment, suggesting that these organisms initially established but later experienced reduced growth due to phage activity. This outcome may be influenced by the anaerobic environment, as anaerobic conditions are known to alter phage–host interactions, including extended latency periods and reduced burst sizes [82]. Future anti-biocorrosion strategies may benefit from advanced bioengineering approaches, such as the deployment of programmable bacteria to autonomously deliver anti-biofilm agents. For instance, recent biotechnological advances have successfully engineered *Escherichia coli* to express functional proteins and deliver them efficiently via outer membrane vesicles (OMVs) [83]. A similar methodology could be adapted to secrete phage-encoded depolymerases directly into SRB biofilms, thereby protecting the enzymes from harsh industrial conditions and ensuring targeted disruption of the EPS matrix.

The use of bacteriophages represents a sustainable and ecologically safe alternative to conventional chemical biocides, but scaling up this technology for field applications requires a rigorous evaluation of its biosafety aspects and ecological implications. Unlike chemical treatments, phages exhibit high specificity and pose no toxicity risks to operators or infrastructure; however, given that SRB play a fundamental role in the global sulfur biogeochemical cycle, the inadvertent environmental release of high viral concentrations could theoretically cause temporary imbalances in non-target natural microbiomes. For large-scale applications, the adoption of mitigation/inactivation strategies is essential before the release of viral particles in industrial effluents. In addition, the commercial application of biological agents in the industry demands robust regulatory frameworks. In Brazil, the recently enacted Law No. 15.070/2024 (Legal Framework for Bioinputs) established specific guidelines for the registration and use of biological control agents. Internationally, bodies such as the Environmental Protection Agency (EPA) in the United States have also adapted their legislation to regulate phages as biopesticides and industrial antimicrobial agents [84]. The global consolidation of these guidelines is a definitive step toward enabling the safe adoption of this technology, ensuring a sound balance between efficient biocorrosion control and environmental preservation.

## 5. Conclusions

The pilot scale experiments demonstrated that a cocktail of non-specific bacteriophages effectively altered the growth dynamics of the mixed sulfate-reducing bacterial culture “AP” and reshaped the microbial composition within the tanks. SEM and profilometry analyses confirm biofilm disruption, reduced surface degradation, and the appearance of distinct structural features, such as filamentous forms. Planktonic microbial community profiling identified 16 predominant taxa across class, family, and genus levels, with associated ASVs linked primarily to fermentative, chemoheterotrophic, and sulfate-metabolizing groups. These trends were consistent with the alpha diversity metrics and highlight the capacity of the phage cocktail not only to suppress the target SRB in the liquid phase, but also to modulate other members of the mixed community, indirectly affecting biofilm dynamics. Future studies are necessary, focusing on the coupon-associated sessile community, an important direction before moving forward to the field-scale steps. Collectively, these findings provide valuable guidance for the development and optimization of phage-based formulations to be integrated in the future in a large-scale storage system and underscore the importance of using broad-spectrum phage formulations capable of targeting complex, multispecies biofilms.

## Figures and Tables

**Table 1:** Characteristics of the lytic bacteriophages composing the cocktail.

Bacteriophage	Host Bacterial Strain	Family/Genus	GenBank Accession No.
vB_EclM-UFV01 (UFV01)	*Enterobacter cloacae* ATCC 13047	*Straboviridae*/*Karamvirus*	ON454249.1
vB_EcoM-UFV09 (UFV09)	*Escherichia coli* 30 *	*Myoviridae/* *Tequatrovirus*	MZ291552.2
vB_EcoM-UFV10 (UFV10)		*Straboviridae*/*Tequatrovirus*	OP555981.1
vB_EcoM-UFV13 (UFV13)		*Myoviridae/* *Tequatrovirus*	KU867876.1

* Strain originally isolated from bovine mastitis and acquired by the Brazilian Agricultural Research Corporation (EMBRAPA).

**Table 2:** Surface roughness (μm) of the carbon steel coupons after 38 days of experiment.

Surface Roughness (μm)	Control Tanks	Treatment Tanks
Arithmetical mean roughness (Ra)	17.51	17.12
Root mean square roughness (Rq)	22.38	21.65
Maximum profile peak height (Rp)	81.67	63.22
Maximum profile valley depth (Rv)	−75.28	−67.36
Total height of profile (Rt)	156.95	130.57

## Data Availability

The authors declare that all data have been included in the article referenced in the article.
